# Reducing Errors in Transition from Acute Stroke Hospitalization to Inpatient Rehabilitation

**DOI:** 10.3389/fneur.2015.00227

**Published:** 2015-10-27

**Authors:** Chloé E. Hill, Priya Varma, David Lenrow, Raymond S. Price, Scott E. Kasner

**Affiliations:** ^1^Department of Neurology, University of Pennsylvania, Philadelphia, PA, USA; ^2^Department of Physical Medicine and Rehabilitation, University of Pennsylvania, Philadelphia, PA, USA

**Keywords:** stroke, rehabilitation, transitions of care, quality improvement, medication errors

## Abstract

**Objective:**

Effective stroke care does not end with acute treatment during hospitalization, but extends through rehabilitation and secondary stroke prevention. In transitions across care environments, stroke patients are vulnerable to errors in communication of diagnosis and treatment. This study aimed to demonstrate that formalized communication between the neurology team and the rehabilitation medicine team would promote secondary stroke prevention and minimize interruptions during rehabilitation.

**Methods:**

The intervention was a standardized verbal handoff by phone between the discharging neurology resident and the admitting rehabilitation resident regarding each patient at transfer. This retrospective cohort study compared a pre-intervention control group (September 2012 to February 2013) and a post-intervention group transferred with the handoff (September 2013 to January 2014). The outcomes measured included errors in communication of stroke severity, stroke mechanism, medications, and recommended follow-up (appointments and tests) as well as emergent brain imaging, return to the acute care facility, and readmission.

**Results:**

The pre- and post-intervention groups were similar with respect to number of patients (50 vs. 52) and demographics including gender (52 vs. 54% female), age (65.8 vs. 64.0 years), severity of illness as measured by the National Institutes of Health Stroke Scale (NIHSS) (10 vs. 6.5), and stroke type (84 vs. 77% ischemic). Implementation of the handoff decreased errors in communication of diagnosis (NIHSS 92 vs. 74%, *p* = 0.02; stroke mechanism 54 vs. 30%, *p* = 0.02). Furthermore, the handoff decreased the proportion with errors in reconciliation of critical medications (42 vs. 23%, *p* = 0.04). However, the intervention did not significantly reduce interruptions of the rehabilitation program, such as emergent brain imaging (8 vs. 12%, *p* = 0.55), or transfers back to the acute care hospital (26 vs. 21%, *p* = 0.56).

**Conclusion:**

Standardized handoffs decreased errors in communication of diagnosis and critical medications for secondary stroke prevention.

## Introduction

State of the art stroke care spans a continuum of care environments, from the emergency room to the stroke unit to the rehabilitation facility and finally home. While expertise at different stages of treatment and recovery has been shown to reduce patient morbidity and mortality (1–3), transitions across these care environments can be hazardous (4).

Following a stroke, there is urgency for stroke patients to participate in early rehabilitation, which can mitigate residual disability (5). However, this goal must be balanced against a patient’s active medical issues, which can interrupt rehabilitation; medical complications occur in 44–96% of stroke patients (6–13) and there is a readmission rate of 3–19% (9–12, 14).

For such medically complicated patients, errors at the time of transfer across care environments are common and may be associated with adverse events and readmission (4, 15). Furthermore, stroke patients are discharged with complicated treatment plans, which vary by stroke mechanism and can require delayed initiation of new medications (such as anticoagulants and antihypertensives) (16). Therefore, the forward communication of a patient’s hospital course and treatment plan at time of hospital discharge to the rehabilitation setting is vital and focused study of effective communication methods is warranted.

This study implemented a standardized verbal handoff between the discharging neurology stroke team and the admitting rehabilitation team. The first aim was to promote secondary stroke prevention care by improving communication regarding the stroke mechanism and improving medication reconciliation of critical medications. The second aim was to minimize interruptions of the rehabilitation program by decreasing emergent brain imaging and decreasing transfers back to the emergency department (ED) and/or readmissions to the acute care hospital.

## Materials and Methods

### Study Design and Subjects

This was a retrospective quality improvement cohort study of stroke patients transferring from the acute care hospital (Hospital of the University of Pennsylvania) inpatient stroke service to the affiliated, but physically and administratively separated, inpatient rehabilitation hospital (Penn Institute for Rehab Medicine). The University of Pennsylvania institutional review board approved this study; informed consent was waived for this study. This study compared a pre-intervention cohort of patients transferred without a verbal handoff from September 2012 through February 2013 to a post-intervention cohort of patients transferred with a verbal handoff from September 2013 through January 2014. The period between these times (February 2013 to September 2013) was used for training of the residents as well as creation and refinement of the verbal handoff and therefore was not included in the analysis. The patients were identified by a database query. If a patient was admitted more than once during the study period, only the first admission for stroke was considered; this resulted in 50 patients in the pre-intervention group and 52 patients in the post-intervention group.

### Data Collection

Data were abstracted for each patient from the acute care hospital and the rehabilitation hospital inpatient electronic medical records. Attention was focused on points of transition: (1) discharge from acute care/admission to rehabilitation and (2) discharge from rehabilitation. The specific documents reviewed were the acute care discharge document, the rehabilitation admission document, and the rehabilitation discharge document; when these records were found to be discordant, further inquiry into the chart was performed. A standardized template was employed to guide collection of neurological diagnosis and stroke type (hemorrhagic or ischemic stroke), the National Institutes of Health Stroke Scale (NIHSS) as a measure of stroke severity (17), stroke mechanism, treatment plan, medication reconciliation on discharge and admission documents, emergent brain imaging during rehabilitation, unplanned returns to the hospital, readmission, and follow-up plan. To ensure consistency in data ascertainment from the electronic medical record, a single reviewer (Chloé E. Hill) completed data abstraction using a standardized data abstraction form (see Supplementary Material). Incorrect data and missing data both were classified as errors in communication. This study was performed under the hypothesis that the intervention would improve the flow of accurate data from the acute care hospital to the rehabilitation hospital. Therefore, if the data were available in the acute hospital care record but not in the rehab hospital record, then this was considered an error because that information did not clearly pass the transition. Errors were tallied from the rehabilitation documents as well as the acute care discharge document.

### Intervention

A standardized verbal handoff was instituted between the discharging acute care hospital neurology team and the admitting rehabilitation hospital team. On the day of transfer, in addition to reviewing the discharge document in the electronic medical record, the admitting physical medicine and rehabilitation resident would call and receive a verbal handoff over the phone. The recommended handoff conversation included discussion of diagnosis, treatment and medications, outstanding studies (either tests completed but not yet resulted or tests planned for the future), and anticipatory guidance about potential short-term problems (see Supplementary Material for details). In the training phase, neurology residents had a reference available to guide them through the recommended handoff conversation. This intervention was piloted in June 2013 and became standard for stroke discharges by August 2013.

### Analysis

The study was powered to detect a 50% reduction in number of patients with medication errors from an error rate of one medication per discharge as seen in a prior survey of stroke discharges from our institution. Comparisons between outcomes pre- and post-intervention were performed using the *t*-test (for age), chi-squared (for gender, stroke type, errors in severity, errors in stroke mechanism, errors in medications, emergent brain imaging, return to ED/hospital, readmission), or Wilcoxon ranked sum test (for NIHSS, co-morbidities, and length of stay). Logistic regression analysis was performed to test associations between errors and return to hospital. Analyses were performed using Stata 12 (StataCorp. 2011. *Stata Statistical Software: Release 12*. College Station, TX, USA: StataCorp LP.). All tests were two-sided and considered significant if *p* < 0.05.

## Results

The two study groups, pre- and post-intervention, were similar with respect to demographics including gender, age, and the number of major medical co-morbidities. Additionally, there were no significant differences in severity of illness as measured by the NIHSS, stroke mechanism, or length of stay in the acute hospital (see Table 1).

**Table 1:** **Demographics**.

	Controls (*n* = 50)	Handoff (*n* = 52)	*p* value
Age (years)	65.8	64.0	0.49
Female sex	52%	54%	0.85
Ischemic stroke	84%	77%	0.90
Intracerebral hemorrhage	14%	21%	0.90
NIHSS^a^ (median, IQR^b^)	10 (6–16)	6.5 (4–15)	0.20
Co-morbidities^c^ (median, IQR^b^)	3 (2–4)	3 (2–3.5)	0.52
Length of stay at acute care hospital (median, IQR^b^)	8.5 (7–14) days	7 (5–12) days	0.09
Length of stay at rehab (median, IQR^b^)	16 (9–22) days	13 (8–19) days	0.08

*^a^Data for NIHSS was missing from the acute care discharge document for 52% of the control group and 44% of the handoff group*.

*^b^Interquartile range*.

*^c^Measured as a count of organ systems with impairment (includes cardiac, pulmonary, renal, endocrine, gastrointestinal/hepatic, genitourinary, hematologic, musculoskeletal, psychiatric, and neurologic systems)*.

**Figure 1:**
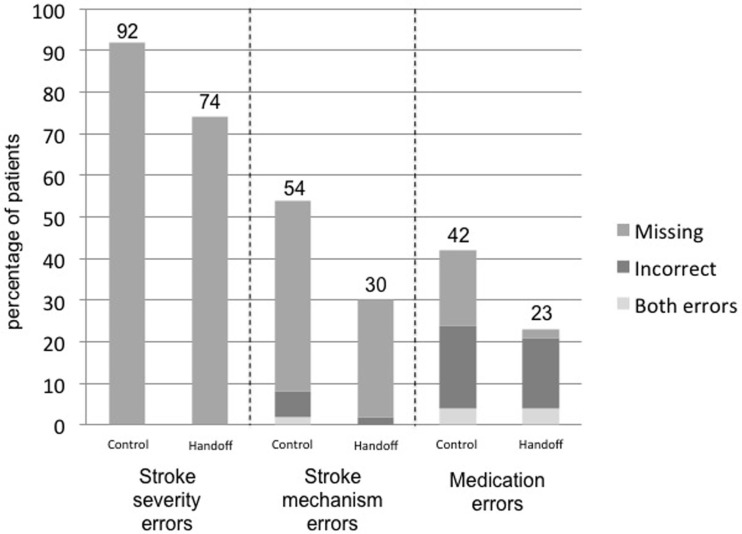
**Types of errors in communication of diagnosis and medications**.

Implementation of the verbal handoff resulted in fewer errors in communication of diagnosis in the post-intervention group, as measured by communication of both stroke severity (92 vs. 74%, *p* = 0.02) and stroke mechanism (54 vs. 30%, *p* = 0.02) (see Table 2). The majority of these errors were due to missing data (not recorded in the medical record) (see Figure 1). Furthermore, the handoff decreased the proportion of discharged patients with errors in the reconciliation of critical medications, i.e., antihypertensive, antiplatelets/anticoagulants, statins, and antiepileptic medications (42 vs. 23%, *p* = 0.04) (see Table 2). Errors in medications included more commonly incorrect medications (such as wrongful continuation of home antihypertensive regimen, incorrect medication dosage, or early initiation of anticoagulation) as well as less commonly missing medications that were intended for the patient but were absent from the medication reconciliation (such as absent aspirin or statin) (see Figure 1); in the case of apparent medication error, further review of the chart was performed to confirm the change was not purposeful.

**Table 2:** **Errors in communication of diagnosis and medications**.

	Controls (*n* = 50) (%)	Handoff (*n* = 52) (%)	*p* value
Stroke severity errors	92	74	0.02
Stroke mechanism errors	54	30	0.02
Medication errors	42	23	0.04

**Figure 2:**
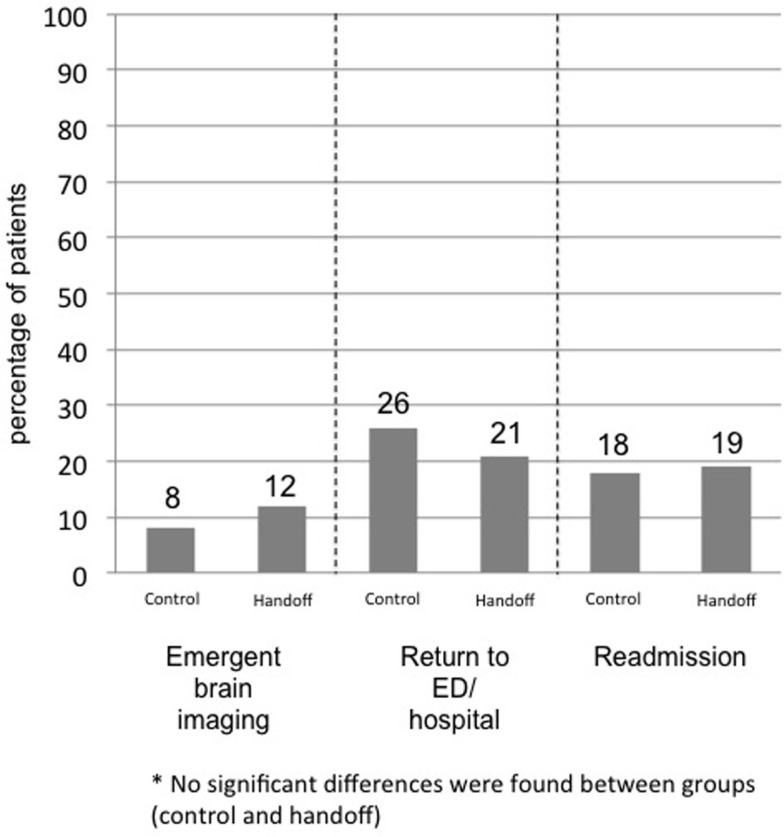
**Interruptions of rehabilitation**.

The intervention did not alleviate interruptions of rehabilitation, such as emergent brain imaging (8 vs. 12%, *p* = 0.55), or transfers back to the acute care hospital/ED (26 vs. 21%, *p* = 0.56). Readmissions to the hospital were high in both groups (18 vs. 19%, *p* = 0.87) (see Figure 2). Return to the hospital was most commonly due to altered mental status followed by tachycardia, anemia, seizure, infection, and headache. Any error in communication was associated with a point estimate suggesting increased ED visits and rehospitalizations, but this did not reach significance (OR 2.2; 95% confidence interval 0.8–6.2, *p* = 0.15). No differences were found between the two groups in errors in follow-up, such as appointments recommended and tests completed (20 vs. 23%, *p* = 0.56).

## Discussion

This study found that a formalized verbal handoff between neurologists at an acute care hospital and the physiatrists at a rehabilitation facility significantly improved communication of stroke diagnosis and reconciliation of critical medications. As both of these have direct bearing on secondary stroke prevention, this handoff could potentially also impact longer-term outcomes, such as recurrent stroke and disability.

The literature suggests that medication errors occur for 49–86% of patients at discharge from the hospital and that such errors can result in adverse events (4, 15). This study found that over half (56%) of the medication errors that were made involved patients’ antihypertensive regimen, which can be confusing because these medications are often held in the acute setting and restarted in the days to weeks after stroke. The next most common type of error was in antiplatelet or anticoagulant medication (26%). Each of these errors represents a major missed opportunity as there is evidence that stroke patients are more likely to continue taking medications which were started during their hospitalization (18, 19). Although errors in recommended evaluation after discharge are also common and have been shown to significantly increase the likelihood of readmission (15), this handoff intervention did not decrease errors in follow-up.

This study also identified a vulnerable population of patients with a readmission rate of 19%, which is comparable to some prior studies (9–12, 14), but is nearly double the rate of readmission for stroke patients discharged elsewhere from our hospital. The intervention did not significantly alleviate interruptions of the rehabilitation program, in keeping with previous studies of transitional care interventions that have failed to improve emergency room visits and hospital readmission rates (20); however, reduction in communication errors could perhaps impact this important outcome in a larger study. These interruptions were characterized and the observed range of chief complaints overlaps with prior studies of stroke patients requiring transfer back to acute medical care (14). As readmissions tended to be medical rather than neurological in nature, perhaps readmissions are not the best measure of the impact of this study’s intervention, which had a primarily neurological focus. In future studies, it may be useful to look at longer-term outcomes, such as recurrent stroke and medication compliance.

There are several limitations to this study, including minimal prior data to inform the study design and a limited sample size to detect a difference between study groups. Additionally, there was non-blinded determination of errors during chart review. Lastly, the analysis made the assumption that the verbal handoff was completed for every patient during the post-intervention study period; if the handoff were not uniformly implemented, then this study would be underestimating its impact. Further, the generalizability of this approach remains to be determined.

While many aspects of stroke care have been extensively studied and protocolized, handoff of stroke patients has been largely neglected despite evidence that a substantial proportion of preventable adverse events in this population are attributable to errors in communication between providers (21). Transition to rehabilitation is especially high risk for communication failure (22). This study demonstrates that this risk can be modified, and the observed improvements are similar to other recently published data supporting a decrease in medical error rates with implementation of a formal verbal handoff program (23). A more structured approach to this handoff might further augment the effect of this intervention.

## Author Contributions

CH – designed protocol, facilitated study intervention, completed data collection, performed statistical analysis, and drafted manuscript. PV – facilitated study intervention and edited manuscript. DL – conceptualized study and edited manuscript. RP – conceptualized study, guided study design, and edited manuscript. SK – conceptualized study, guided study design, performed statistical analysis, and edited manuscript.

## Conflict of Interest Statement

This research was conducted in the absence of any commercial or financial relationships that could be construed as a potential conflict of interest. No payment or services from a third party were received for this work. The authors have no financial relationships to disclose.
