# Supplementary material for: Reducing Errors in Transition from Acute Stroke Hospitalization to Inpatient Rehabilitation
Source: Front Neurol. 2015 Oct 27;6:227. doi: 10.3389/fneur.2015.00227 (PMC4621425; doi:10.3389/fneur.2015.00227)
Supplement: Supplementary file 1 [file data_sheet_1.pdf]

Appendix
The verbal handoff occurred for each patient on the day of discharge. The accepting rehabilitation resident called the discharging neurology resident to complete the handoff conversation. During the training phase, neurology residents were introduced to the following checklist in an educational session and copies of the checklist were made available as references to guide the handoff conversation. Additionally, the discharging neurology resident was encouraged to elicit and answer any further questions from the accepting rehabilitation resident.

**Discharge to Rehab**<br>**HANDOFF CHECKLIST**
o Vitals
o Exam
o Diagnosis & etiology of stroke
o Work-up pending
o Antihypertensives (plan for permissive hypertension), antiplatelet/anticoagulant agent (INR goal if appropriate), statin
o Cardiac arrhythmia?
o Does inpatient med list match discharge summary med list?
o DVT prophylaxis
o Agitation or recent one-to-one
o Lines/tubes (service and date placed)
o Neurology and specialist follow-up

Of note, completion of the handoff was not documented in the chart and not reviewable for this study.

The following template was used for data abstraction:

STUDY ID:	*Age:*<br>*Gender:*		
	**ACUTE CARE DC DOC**	**REHAB ADMIT DOC**	**REHAB DC DOC**
STROKE DIAGNOSIS:	Diagnosis:<br><br>NIHSS:<br><br>Mechanism:	Diagnosis:<br><br>NIHSS:<br><br>Mechanism:	Diagnosis:<br><br>NIHSS:<br><br>Mechanism:
STROKE CARE PLAN:	Blood Pressure:<br><br>Antiplt/Anticoag:<br><br>Statin:<br><br>Antiepileptic:	Blood Pressure:<br><br>Antiplt/Anticoag:<br><br>Statin:<br><br>Antiepileptic:	Blood Pressure:<br><br>Antiplt/Anticoag:<br><br>Statin:<br><br>Antiepileptic:
MEDICAL CO-MORBIDITIES:			
Length of Stay:			
Pending Workup:			
Follow-Up Plan:			
Brain Imaging in Rehab?			
Return to Acute Care? Chief Complaint?			
	**ACUTE CARE DC MED REC**	**REHAB ADMIT MED REC**	**REHAB DC MED REC**
MEDICATIONS:			
